# On the Classification, Administration, Modus Operandi, and Combination of Medicines

**Published:** 1834-04-01

**Authors:** J. Stevenson Bushnan

**Affiliations:** Surgeon to the Dumfries Dispensary, &c.


### ORIGINAL COMMUNICATIONS.

### *On the Classification, Administration, Modus Operandi, and Combination of Medicines.* By J. Stevenson Bushnan, f.l.s., Surgeon to the Dumfries Dispensary, &c.

### 1. *Classification of Medicines in general.*

The medicines commonly employed in the treatment of diseases are derived from all the three kingdoms of nature, and may be conveniently classed according to the particular effects which they in general produce; while these classes may be further arranged in the order of the organs on which they appear severally in general to act. The chief classes of medicines commonly enumerated are the Errhines, acting principally upon the nostrils; the Sialogogues, upon the salivary glands; the Demulcents and Expectorants, upon the lungs; the Emetics, upon the stomach; the Purgatives and Carminatives, upon the intestines; the Diuretics, upon the kidneys; the Emmenagogues, upon the uterus; the Diaphoretics and Epispastics, upon the skin; the Astringents, upon any of the foregoing organs: and the Stimulants, Tonics, Antispasmodics, Sedatives, and Narcotics, upon the spinal marrow and brain. The Antacids and Anthelmintics, which act, the former in neutralizing acid matters in the stomach, and the latter in destroying intestinal worms, cannot, with propriety, be referred to any particular organ.

The above classification of medicines, although convenient for practical purposes, and to a certain degree precise, is at the same time quite artificial, and most indefinite and arbitrary. To say nothing of the long and violently agitated question respecting the primarily stimulant or sedative operation of certain medicines, and which, according to the different views which any two persons might choose to take of it, would appear to justify them in placing the same medicine under two diametrically opposite classes, there are numerous substances, such as ipecacuanha, assafœtida, digitalis, opium, antimony, and mercury, which, without any sophistry, may with equal propriety be referred to almost any class. Thus, ipecacuanha, according to the quantity in which it is given and other circumstances, is either an expectorant, an emetic, a purgative, a diuretic, a diaphoretic, a tonic, or an antispasmodic; and opium (though it belongs neither to the class of purgatives nor astringents,) may be given to fulfil, not only very different but directly opposite indications, as that of opening the bowels in colic, and of binding them in cholera. Alum also, (a generally admitted astringent,) when given in large doses, frequently purges; and something similar may be said of almost every individual medicine; but it will sufficiently appear from these examples how abortive must be every attempt to make any thing like a precise classification of medicines on such principles, and how essential it is therefore to study the action of each in detail, without being much influenced by the place which it occupies in generalizations which nature refuses to acknowledge.

But, imperfect as these principles confessedly are, they are perhaps the best which the nature of the subject will admit, and certainly much better than those formerly adopted.

By the ancient physicians, the several articles of the materia medica were spoken of, in conformity to the system of pathology then in vogue, principally according to their supposed virtues of determining the fluids to and from certain parts, of promoting the desired concoctions of morbid matters, of drawing abscesses to an head, or evacuating their contents, of stopping hæmorrhages, uniting wounds, cleansing and healing ulcers, and so forth; and, even after the revival of letters, the same views prevailed among the early writers on the materia medica. At that time the majority of diseases was attributed to an "ἀκρασια," consisting in an inordinate flow to certain organs of the one or other of the four principal fluids of the body, the object of which was supposed to be to promote the concoction and subsequent expulsion of certain crude morbific matters, imagined to be collected in these organs, against which, as against an invading power, these fluids were detached by the conservative principle of the body. Hence arose the well-known axiom "ubi irritatio, ibi fluxus;" and it was only, or chiefly, for the purpose of co-operating with these supposed salutary operations of nature, that medicines in general were formerly administered.

These views alone prevail in the writings of Bauhin, Columna, Fallopius, and the other early writers on the materia medica in modern times; nor was the system of things much improved by the addition of the antalkaline and antacid remedies of the chemists, or the inspissants and attenuants of subsequent pathologists. It was only gradually that medicines came to be arranged upon the principles now commonly adopted; the writings of Boerhaave, Gaubius, and De Gorter, and even those of Cullen, Vogel, Murray, Young, and others, being still considerably adulterated with the notions so long before prevalent on the subject; but it is still chiefly to these authors that we owe the establishment of the principles which, imperfect as they are, are perhaps, as I have already said, the best of which the subject is susceptible. How utterly idle, however, must be every attempt at a perfectly precise arrangement of medicines "after their kinds," upon the basis of their supposed effects upon the body, is sufficiently obvious, not only from the nature of the subject itself, but from the discordant, unnatural, and unintelligible systems which many of the labourers in this visionary vineyard have left behind them. "Des moyens identiques," says Bichât, "ont eu souvent des noms différens, suivant la manière dont on croyoit qu'ils agissoient. Deobstruent pour l'un, relâchant pour l'autre, refraîchissant pour un autre, le même médicament a été tour à tour employé dans des vues toutes différentes, et même opposées."

2. *Administration of Medicines in general.*

Medicines, whether solid, liquid, or aëriform, may be administered by numerous different avenues. They may be either snuffed up by the nostrils, or applied to the eyes in the manner of collyria, or to the mouth by chewing, gargling, fumigating, rubbing into the gums, &c., or inhaled by the lungs, or even injected into them by an opening made into the trachea; or they may be swallowed, or injected into the rectum as a clyster, or inserted into it as a suppository, or injected into the urethra or vagina; or they may be applied to the skin in the form of fomentations, poultices, lotions, baths, &c., or introduced through the skin by friction, or by continual contact; or, lastly, they may be applied to a blistered surface, or inserted into a wound, or injected into a vein.

The most ancient way of administering medicines is probably by the skin, to which they were applied in combination with oils, the external use of which was in the primeval ages so universal. The custom, also, so common among the ancient Egyptians, and their successors, of burning resinous and aromatic substances, as well in their religious ceremonies as for the purpose of purifying the air, would soon give rise to the use of fumigations by the mouth, and inhalations by the lungs. We find that, so early as the time of Homer, the vapours of sulphurous acid were in common request for purifying the air after sickness and death; and, during the celebrated plague of Athens, (430 years before Christ,) we are told that strong perfumes and immense fires were used as a chief prophylactic remedy; and it is to these practices that we must ascribe the early use, both by Egyptians and

Greeks, of the vapours of bitumens, pitch, sealing-wax, sulphur, and similar substances, in diseases not only of the fauces, but of the lungs; a practice revived in 1664 by Bennet, and continued by Willis, Mead, and many others, down to our own times. Similar fumigations were formerly often employed by the vagina as emmenagogues. The administration of medicines by the mouth is said to have been suggested to the ancient Egyptians by dogs and apes; the former having been observed to take grass as a vomit, and the latter the pulp of cassia as a purge, as often as their constitutions required these reliefs; and the first use of clysters among them is reported by Herodotus to have been merely an improvement upon the practice of the ibis, which is in the habit, when in the water, of clystering itself with its long beak. The use of masticatories, or local sialogogues, has from time immemorial been prevalent in all warm climates as a luxury—sometimes the betel leaf or areca nut, at others quicklime, being employed in this way—and such substances, therefore, would of course be sometimes used medicinally; and, although we do not hear of the habitual use of any kind of snuff before the introduction of tobacco, (and indeed the old prejudices against blowing the nose were incompatible with such a practice,) we know that errhines, and some antispasmodics, administered by the nostrils, were favourite remedies with Hippocrates. In like manner, gargles, suppositories, injections by the vagina, and medicinal pessaries, (a method of administering medicines at present almost entirely discontinued,) as well as baths of medicated waters and oils, and general fumigations of the body with the vapours of sulphur and various other substances, as recently recommended by Dominiceti, Gall, and others, were in common use among the ancients, together with certain other practices, such as that of pouring medicines into the ears, which is mentioned by Aretæus and Cælius; "quo per sensuales vias ad membranas cerebri recorporativa virtus adveniat," and the memory of which Shakspeare has immortalized:

—— "Sleeping within mine orchard,
My custom always in the afternoon,
Upon my secure hour thy uncle stole,
With juice of cursed hebenon in a vial,
And in the porches of mine ears did pour
The leperous distilment."—*Hamlet*, act i. scene 5.

Perhaps, therefore, the chief, or only new methods of administering medicines, are by friction upon the skin, injection into a vein, friction upon the gums, injection into the lungs by the trachea, insertion into a wound, and application to a blistered surface. Of these, the practice of introducing medicines by friction upon the skin seems to have originated with James Berenger de Carpi, who about the year 1520, for the first time, administered mercury in this way in the cure of lues venerea; and the same means of administering scammony, jalap, squill, rhubarb, opium, and other medicines, has been since employed by Spallanzani, Chiæranti, Vacca, Berlinghieri, Brera, Alibert, Pinel, Dumeril, and others. The origin of injecting medicines into the veins seems to have been nearly coeval with that of the transfusion of the blood of one animal into the veins of another, which was recommended by Libavius, of Halle, in 1615, and practised, probably, for the first time by Lower, in 1665; although many others have put in their claims as the institutors of an art from which so much was at first expected. Lower, however, practised on brutes only; the first to perform transfusion on man having been Denys and Emerey in France, and Riva and Manfredi in Italy; but the results having been in most cases discouraging, the practice was forbidden, as well by the parliament of Paris, as by the Pope, and though since brought again into notice in Switzerland by Dumas and Prevost, and in this country by Leacock, Blundell, Waller, and others, it has not yet quite recovered its reputation. The first to inject medicines into the veins is said to have been "mathematicus ille insignissimus," D. D. Christopher Wren, about the year 1656; the subject was farther prosecuted by Boyle, Oldenburgh, Fracassati, Frabricius, and Dr. Timothy Clark, between this period and 1678, the last of whom tells us that he had administered with success "emetica, cathartica, diuretica, et opiata isto modo:" and it was proved, lastly, by John Hunter, that the effects of ipecacuanha, jalap, opium, &c., so administered, were more certain and speedy than when received into the stomach. The method of administering medicines by friction upon the gums was employed perhaps for the first time in 1811, by Dr. Chrestien, with his preparations of gold, &c., and subsequently by Mr. Clare, with calomel; and that of injecting them into the lungs by the trachea, proposed for the first time by Goodwyn, has been recommended lately by Mayer, Autenrieth, and Schlaeffer; and the great celerity with which absorption takes place from this organ is certainly in favour of the practice. Lastly, the method of inserting medicines into a wound was followed with the corrosive sublimate in the cure of lues venerea by Mr. Clure, in 1819; and that of applying them to a blistered surface seems to have been proposed first by Lesieur, of Paris, within these last few years, and has been followed with success by Dr. Martin, and many others.

### 3. *Operation of Medicines in general.*

Various as are the avenues by which medicines may be received into the body, it is very remarkable that, in whatever way a medicine is so introduced, its operation is still for the most part—like that of a poison, or any other exciting cause of disease—upon one and the same organ. Thus, mercury is an equally certain sialogogue, in all the various ways it is administered; the demulcents, expectorants, diuretics, and most other classes of medicines, act equally specifically on the mucous membranes, kidneys, &c., whether they be received into the stomach or introduced through any other channel; tobacco and white hellebore act as emetics, when either applied to the skin or introduced by the anus; black hellebore, colocynth, gamboge, antimony, &c., act as purgatives, when either snuffed up by the nostrils, or applied to the eyes in the manner of collyria, or handled for a considerable time, or inserted into an issue, or mixed with blistering plaster; and cantharides acts as an astringent when either swallowed or applied to the skin: and that the specific effects of almost any medicine may be produced by injecting it into a vein is at present very generally known. It follows from these facts, that, in explaining the mode of action of medicines in general, we ought to dwell, not so much on the means by which they reach the organ on which they act, as on the specific nature of the stimulus which they convey, and which, whatever be the channel by which it be conducted, produces nearly the same effects. It has been known from the most ancient times, and the works of modern physiologists have inculcated the fact with a considerable degree of precision, that, though the whole body is endowed with a general irritability, or susceptibility of impressions from foreign agents, each individual organ of the body has a peculiar kind of irritability of its own, adapting it to be acted upon by certain stimuli more remarkably than by others. If this were not true, it is obvious that the same stimulus should produce in every organ of the body the same effect; which is so far from being the case, that there are hardly any two organs of the body which precisely agree in this particular. Thus, arterial blood is the natural stimulus to the left, and venous to the right cavity of the heart; the alimentary matters and their products are the natural stimuli to the stomach and intestines; and the several secreted fluids are the natural stimuli to the respective ducts and receptacles by which they are passed, or in which they are allowed to accumulate; and, moreover, the deleterious effects of any one of these substances in any organ but that specifically adapted for its passage or reception, are a conclusive proof of the different kind of irritability which each enjoys.

The doctrine of specific kinds of irritability, I have said, is not new, although it is only lately that this doctrine has been applied with any precision to the explanation of the action, as well as the exciting cause of disease, as of the remedies employed in removing them.

The several genii which the most ancient philosophers supposed immediately to preside over the function of each organ of the body, and the peculiar δυναμις which Hippocrates and Galen conceived to be resident in each, distinct from and subservient to the φυσις, ενορμον, πνευμα, &c. (by which terms they signified the universally presiding spirit of the body,) were manifestly the same thing expressed in a more vague manner; and the admission by the earliest physiologists of a pulsating power in the heart, a concocting power in the stomach, and so forth, was neither more nor less than an acknowledgment of the specific kind of irritability with which each of those organs was endowed. The same thing was understood by Van Helmont under the name of *Archæii insiti*, the number of which almost equalled the organs of the body, though all were held in subordination by one sovereign *Archæus*, corresponding to the πνευμα, &c., already mentioned, and supposed to hold his court in the stomach. Harvey also admits in each organ a *sensus proprius*, subject to the general *anima* by which the whole body was actuated; and Glisson speaks of each organ as possessed of, in addition to its general irritability, a "*spiritus regens*, qui aliud in jecore, aliud in liene, aliud in pancreate, aliud in ventriculo et intestinis operatur."

The subject of these *imperia in imperio* was further prosecuted by Bordeu, the commonly reputed father of modern French physiology, who distinctly propagated the doctrine that it was owing to such a cause, "que chaque organe sent et se meut à sa manière;" and who certainly suggested to Bichât all that the latter subsequently did in assigning to each organ a specific irritability, by which its peculiar function, or *vita propria*, forming a part of one or other of the two heads of functions, improperly called lives, carried on by the body in general, was effected. In this respect, then, modern authors, as remarked by Dr. Barclay, "instead of advancing anything new, as they probably supposed, have only revived one of the most rude and antiquated notions in all physiology." Now, it can hardly be questioned that it is owing to this peculiar susceptibility in certain organs of certain impressions, that particular exciting causes of disease, such as specific contagions, poisons, and so forth, in whatever manner they be applied to the body, produce always particular effects; and precisely the same explanation, as it appears to me, must be given of the equally specific action of all medicines, in whatever manner they be administered. Not only every class of medicines, but every individual medicine, may be with great reason presumed, like every other agent on the body, whether salutary or deleterious, to afford a stimulus more or less distinct from that afforded by any other. When applied to the body therefore, in any way, each will be comparatively inert with respect to all those organs to the peculiar irritability of which this specific stimulus is not adapted, and will act only, or chiefly, on those which are thus calculated to receive it; and this equally, whether the medicine in question operate directly on the organ, or being applied to some other one, produce there such an irritation as, conveyed by certain means to the organs on which it is more properly to act, bring about its specific effects; in other words, operate by sympathy. It would involve me in a long and very intricate question to investigate the means by which these sympathetic irritations are effected. The existence of such a power resident in the body is unquestionable, whether we can explain its nature or not; and the innumerable instances of increased secretions and other inordinate actions, very analogous to the effects of certain medicines, produced by mere sympathy, without any suspicion of an altered condition of the blood or any other change, seem to justify us in supposing when medicines are applied otherwise than directly to the organs upon which they are to act, though they may sometimes be taken up by the blood, and thus operate in the same way as when injected into a vein, yet that they may, and usually do, produce their effects in the former way alone.

The notions of the ancients with respect to the mode of action of medicines are unworthy of being remembered, except as examples of the absurdities into which men are liable to be betrayed by a close adherence to favourite systems. It had been established that of four elements, each distinguished by a certain essential property, as dryness, moisture, coldness, and heat, everything in nature was composed; that of certain definite combinations of these elements were formed the four principal fluids of the body; and that an *ακρασια*, or preternatural local accumulation of one or other of these constituted disease in general. Now, in repairing this loss of balance in any part of the fluids, attended of course by their essential properties, it followed that substances must act either by abstracting such properties as were superabundant, or by supplying such as were deficient; and hence arose the hypothesis that every medicinal substance which was a remedy for dryness was moist, for moisture dry, and so forth; and it might be either the one or the other, and at the same time either cold or hot, and every one of all these in either the first, second, third or fourth degree. Moreover, it was the leading or cardinal property of the medicine which determined its sensible operation; emetics, for instance, going upwards because they were hot, seeing that it was the nature of heat to ascend, and purgatives downwards because they were cold, each attracting forth with it such humours as were most allied to itself.

These doctrines, after the revival of literature, gave way to views respecting the action of medicines, in general either strictly chemical, or exclusively mechanical, the living body being spoken of as an inert mass, and medicines being described as operating, each in its own way, either according to its particular affinities, or, as they were then called, sympathies or antipathies, on the one hand, or, according to its bulk, or the size and form of its particles, on the other; and it was not till after Hoffman had shewn that the causes of diseases operated always on the nervous system alone, that the action of medicines also began to be referred to the same system. Still, however, much of the old chemical and mechanical leaven continued to prevail in the common hypotheses on this subject; and this equally, whether a medicine were brought into immediate contact with the organs affected, or reached them by means of the blood, which for a long time seemed to be the only two conceivable methods by which it could affect them. Thus a common explanation of the salivation produced by mercury was, that the medicine, taken up by the blood, passed directly upwards, owing to its great weight, which disposed it to preserve a straight line as transmitted by the heart; or that it did so by its admixture with the blood, which was thus so far attenuated as to be strained through the pores of the salivary glands like so many sieves, which was the explanation of Van Swieten; or that it operated by combining with the supposed ammoniacal salts of the blood, and thus attenuating it, as supposed by Cullen: or lastly, that it passed off by the salivary glands, because the secretion of the kidneys, containing phosphoric acid, which forms with mercury an insoluble compound, refused to transmit it, which was the hypothesis of Murray. The action of mercury also in curing lues venerea was generally represented as that of neutralizing the supposed poison in the blood. In like manner, a common explanation of the effects of astringents was, that they increased the cohesion of the fibres of the parts to which they were applied by a species of tanning; of that of refrigerants, that they produced cold in proportion to the rapidity with which they were dissolved, owing to their increased capacity for caloric; of that of iron as a deobstruent, that, being seven times heavier than vegetables, it was seven times better adapted to force its way through any obstructions; and of that of antispasmodics, that, operating upon the heart as a simple stimulus, they produced so increased a flow of blood through the whole body, that the local congestions, upon which spasm was supposed to depend, were thereby, as it were, washed away! When a medicine was neither directly applied to the organ upon which it acted, nor could be conveniently supposed to be taken up by the blood, it was either stoutly denied that it possessed the power attributed to it, or was conceived to operate by contiguity; and it was upon the principle of the contiguity of the heart and stomach that the effects of antispasmodics just mentioned, of that of the lungs and stomach, that those of expectorants, and of that of the uterus and rectum, that those of emmenagogues, were generally explained. At other times, however, still greater ingenuity was called forth; as in ascribing the effects of local sialogogues to the compression of the salivary glands during their mastication, and those of demulcents to their smearing the surface of the larynx during their passage into the stomach, so that, the frequency of the cough being thus diminished, more time was allowed for the inspissation of the secreted mucilage; and it is only very lately that the effects of those purgatives which operate chiefly on the small intestines have been attributed to their more rapid solution, and those of such as operate chiefly on the large intestines, to their more tardy solution in these organs.

But how extremely vague and inconsistent most of these notions are, and how irreconcileable some of them are to the fact, that certain medicines operate, for the most part, on a certain organ, and in a certain manner, by whatever avenue and in whatever form they are introduced, must be abundantly obvious. The luminous doctrines of recent pathologists respecting the nature of those changes which constitute disease, aided by those of Bordeu with respect to specific irritabilities, and by the general observation of the immense extent of sympathies, by which certain irritations of almost any one part may be conveyed to almost any other, have introduced in general much juster views of the action of medicines in curing diseases; and, though the experiments of

Magendie, Brodie, Orfila, Christison, and others, have rendered it probable that many, if not all medicines, as well as poisons, *may* be taken up by the blood, and thus indirectly produce their effects, such a circuitous mode of action seems to be in general by no means more essential to the action of any medicine than to that of any exciting cause of disease. As a matter of curiosity it may be observed, that Dr. Paris enumerates, in capital letters, four "distinct and different modes" in which he imagines medicines may act: first, by "actual contact," including the conveyance of the matter by either the veins or lymphiferous and chyliferous vessels, and whether received by absorption or by a wound, both which however he calls absorption; secondly, by "an impulse conveyed by the instrumentality of the nerves; thirdly, by "the sympathetic control exerted by the stomach on distant parts;" and, fourthly, by "the operation of contiguous sympathy." But, to say nothing of all these modes being unquestionably resolvable into the action of medicines upon the nervous system alone, if in the two last modes they do not produce "impulses," and these be not conveyed "by the instrumentality of the nerves," it would be gratifying to know what they *do* produce, and how their action *is* conveyed; and if they be in both respects similar, it would be satisfactory to know what was the use of enumerating them as modes "distinct and different" from the second and from one another. But this arrangement of the common modes of action of medicines in general, is nothing to what Dr. Paris has attempted to establish with respect to that of each particular description of those medicines, the sensible effects of which are the same, arranging the individuals upon this airy basis into classes, orders, genera, and species, with as much precision as if we really knew anything at all about the matter, and as if all such speculations were not, as he himself calls some of the theories of his predecessors, "philosophical webs which ingenuity has woven for us, the device of which is beautiful, but the fabric too frail to endure the touch."

It must be sufficiently obvious to every reflecting man that the present state of science does not justify us in affecting to arrange medicines, any more than exciting causes of disease, according to their modes of action; and it will be well if the judicious remarks of M. Cap and others, recently made on this subject, put a stop, perhaps for many years to come, to all attempts of the kind.

A. *Operation of Evacuant Medicines.*

The effect of all medicines by which discharges are promoted, such as errhines and sialogogues, seems to be that of stimulating preternaturally, in the first instance, the capillary arteries of the organs whence the evacuation is to proceed; and this inordinate irritation being succeeded, sooner or later, by a corresponding collapse of these arteries, a state analogous to inflammation, and a consequent increased or altered secretion, are the results. It is sufficiently well known, however, that the quantities of medicines required for producing evacuations are considerably greater in cases where the agency of the brain is either suppressed or perturbed, as in sanguineous apoplexy, catalepsy, insanity, and tetanus; the constant stimulus derived from this organ being apparently always conducive, and sometimes almost essential to their operation; and it has accordingly been observed, by Mayo and others, that the action of such medicines may in general be suspended in the lower animals by compressing the brain. It was formerly a much-agitated question, as I have before remarked, whether the action of these medicines, as well as medicines in general, were primarily stimulant or primarily sedative; but this question has for some time past less occupied the attention of therapeuticians. It can hardly be disputed that every positive agent upon the body, and consequently every medicine, is primarily a stimulant, although the more permanent operation of some, and perhaps their only sensible operation, may be that of a sedative. This was the doctrine first of Tralles with respect to the narcotics, and afterwards of the celebrated Dr. Brown with respect to all medicines; but from this doctrine the modern Brunonians, as Rasori of Milan, Tommasini of Bologna, and Borda of Pavia, recede, contending that some medicines (which they call, not sedatives, but contra-stimulants,) act directly in repressing action; and the same opinion is maintained in Germany, by Horn and others, with respect to their negative stimulants. It is probable, nevertheless, that Brown was right; but the subject is not worth half the trouble that has been bestowed upon it, since it is the permanent, not the temporary or primary effect of a medicine that we have any real interest in understanding. I have said that the collapse of the capillary arteries of those organs from which an increased discharge is to be produced constitutes a state analogous to inflammation; and it is well known that the secretions of every part are, *cæteris paribus*, in the direct ratio of the quantity of blood which the capillary arteries of this part contain. Hence has arisen the prevalent notion that the action of evacuant medicines is to produce increased determinations of blood to those organs upon which they operate. It is not improbable, however, that the whole doctrine of increased action of the larger arteries, by which alone such determinations could be produced, is founded in error, and supported by prejudice; and that not only the increased secretions, but blushing and the turgescence of certain organs, and the violent pulsation of the arteries leading to them, on the one hand, and the paleness and flaccidity of these organs on the other, imply not a greater or less action of the larger arteries, but, on the contrary, a less or greater action of their capillary extremities, by which they either retain for a longer time, or expel more rapidly, the blood which they receive. There is no cant in all pathology more common, and at the same time more totally unsupported, than that of increased determinations of blood; since, even admitting the muscularity of the larger arteries, (which is a very questionable point,) although it is easily conceivable how this might have the effect of excluding blood from certain organs, it is quite inconceivable how it should have the effect of determining blood to them. The capillary arteries of each part, however, have obviously an action independent of that of the heart; and it is consequently much more reasonable to believe that the greater quantity of blood collected in each organ, preparatory to an increased secretion, depends not upon more being carried to them, but upon what is carried being less immediately propelled forwards. Nor is the increased pulsation of the larger arteries under similar circumstances any argument against this doctrine. Such an increased pulsation may be immediately produced in the digital arteries by tying a ligature tightly round the extremity of the finger; and in this instance it evidently indicates not a greater determination of blood to the part, but a less easy transmission of blood through it; and, when a similar pulsation occurs spontaneously, it seems infinitely more satisfactory to presume that it is the capillary arteries which are the first cause of this perturbation, and that the larger arteries appear to act more forcibly only because the blood has a greater resistance to overcome: in the same way as a rivulet, which flows placidly along the rest of its bed, beats with violence against any impediment placed in its course, although the propelling powers are no greater here than elsewhere. But, independently of the improbability of such increased determinations of blood ever taking place, they are obviously quite unnecessary in the instances of increased secretions; every organ, even in the natural state of its functions, receiving perhaps two or three hundred times more blood than is employed in this process. The kidneys, for example, are estimated to receive about 450 pounds of blood during the twenty-four hours, while the secretion of urine does not commonly exceed thirty ounces. Is it necessary to imagine that there must be an increased determination of blood to these organs, to explain the operation of a diuretic medicine, upon the presumption that the 450 pounds, just mentioned as their daily allowance, cannot of themselves spare half a dozen ounces more than usual to be turned into urine? The action of these, as well as all other evacuant medicines, is evidently upon the capillary arteries of the part, to the specific irritability of which they are adapted; and the accumulation of blood in this part, necessary to increased secretion, is owing to the diminished action of these arteries; a necessary consequence of the increased irritation to which they have been exposed.

B. *Indications which they fulfil.*

Evacuant medicines are employed sometimes, as the purgatives, emmenagogues, and diaphoretics, in order to promote the natural secretions by any means impeded; at others, as the demulcents and expectorants, in order to change the nature of local secretions; at others, as the emetics, purgatives, and carminatives, in order to relieve certain organs from a foreign load; and at others, lastly, as the errhines, sialogogues, emetics, purgatives, diuretics, and diaphoretics, to operate by revulsion: in other words, when an organ upon which we cannot immediately act is affected with inflammation, consisting in a preternaturally relaxed state of its capillary arteries, to bring about in some distant part a new irritation, which, being conveyed by sympathy to the organ primarily affected, may stimulate its vessels to a healthy contraction. This is not the ordinary explanation, but I feel satisfied it is the true one, of revulsive remedies. The doctrine of revulsion is as old as Hippocrates; and the attempt to cure inflammatory and other diseases by promoting evacuations from distant organs was founded on the observation of the critical discharges by which such diseases seem to be often suddenly resolved, or which, at least, often attend their resolution. Of course, the benefit derived from the practice was at first explained by the theory of a translation of peccant matters, or of the fluids called into play in order to expel them; but, as it was soon noticed that remedies called revulsive frequently produced equally good effects, although unattended with any discharge, it became necessary to establish it as a law of the animal economy, that two morbid impressions could not subsist together in the same body, so that, a secondary and stronger one being promoted, the primary and weaker one necessarily ceased, "Δύω πόνων," says Hippocrates, "ἅμα γινομένων, μὴ κατὰ τὸν ἀυτὸν τόπον, ὁ σφοδρότερος ἀμαυροῖ τὸν ἕτερον;" and this assertion, gratuitous as it certainly is with respect at least to irritability, which has, as I have shewn, many totally independent mansions, however well founded it may be with respect to sensibility, which has but one, has been received since his time almost as an axiom; and while inflammation was considered to consist in increased action of the part affected, it was certainly a very convenient one. But inflammation is at present generally understood to consist, not in increased, but in diminished action of the capillary arteries of the part; so that our explanation of the benefit to be derived from revulsive remedies must be, *toto cœlo*, different from that formerly adopted, and must be founded upon the presumption that they communicate, not abstract a stimulus, and thus promote by the contraction of the dilated vessels, already, if necessary, freed by bloodletting of a part of their load: and this doctrine, however staggering it may be found, as directly opposed to preconceived opinions, is perfectly reconcileable to every unprejudiced view of the question, and either must be adopted, or the whole modern theory of inflammation must be entirely abandoned. The action also of those direct applications found most efficacious in the cure of inflammation is highly favorable to this view of that of indirect or revulsive remedies. "When such inflammation occurs in a superficial part, as the eyes, tonsils, or skin, the local applications in the forms of collyria, gargles, and lotions, from which we derive most benefit, are such as immediately irritate the dilated arteries: and, in the inflammation of a deep-seated part, some of the most efficacious local remedies, such as heat, electricity, and acu-puncture, manifestly act as direct and powerful stimulants;" and the only difference that seems to exist between the operation of these remedies and those reputedly revulsive is, that, what the former effect directly, the latter effect indirectly, or by sympathy.

### c. *Operation of Astringents.*

Directly opposed in their sensible effects to the evacuant medicines are the astringents, the peculiar action of which seems to depend upon the constriction of the capillary arteries which they occasion, being generally (as blushing sometimes immediately succeeds paleness,) followed immediately by a proportionate collapse; and this again (as a more or less permanent paleness frequently succeeds a blush,) by a more or less permanent constriction. That this is the case is rendered probable by the fact, that some of the strongest astringents, such as alum, if given in too large quantities to allow of this secondary constriction, prove, as I have before observed, not astringent, but relaxing. It must not, therefore, excite surprise that this class should contain many of the medicines commonly enumerated among the evacuants; since it may be easily inferred, from what has already been said, how slight a variation of circumstances may make the same medicine give rise, at different times, to directly opposite effects.

### D. *Operation of Stimulants, Sedatives, &c.*

The stimulants and tonics appear to be analogous in their action to the several evacuant medicines already spoken of; and the sedatives, antispasmodics, and narcotics, to the astringents; inasmuch as the two former seem to produce, first a constriction, and afterwards a dilatation of the capillary arteries of the brain, and, consequently, first a diminished, and afterwards an increased evolution of the influence derived from this organ; and the three latter, in like manner, seem to produce, first, a diminished, afterwards an increased, and, lastly, a still more diminished evolution of this influence. The stimulants seem to differ from the tonics principally in their effects being more sudden and violent, and in the same degree more transitory, since both act apparently in exciting equally every organ of the body. Of those classes of medicines, however, which seem to lessen the action of the brain, those are called sedatives which diminish the stimulus sent from this organ, more particularly to the heart; those are called antispasmodics which diminish the stimulus sent from the brain to any of the muscles, whether of involuntary or voluntary motion; and those are called narcotics which at the same time diminish sensibility, the faculty of thinking, and the power of exciting voluntary motion.

### E. *Operation of Antacids, &c.*

The two last classes of medicines which I have mentioned are the antacids and anthelmintics. Of these, the action of the former is directly chemical; while that of the latter is either chemical, as operating in dislodging the worms by dissolving the mucilage in which they are imbedded; mechanical, as operating by friction; or poisonous, as operating by directly killing them. Simple purgative medicines also frequently act as anthelmintics, and are commonly used to assist the action of those substances more properly so called; but to the operation of these medicines, as evacuants, I need not again revert.

### 4. *Combination of Medicines.*

Medicines, in order to adapt them to the various methods in which they are administered, are prepared, either separately or conjointly, in various forms, solid and liquid; but, in prescribing compound medicines, it is proper continually to keep in mind that the properties of the compound are by no means always the mean of the properties of the ingredients, but that, while sometimes these latter properties are corrected or neutralized by such combinations, at others they are considerably increased, and at others entirely new properties are superadded. It is sufficiently well known that two or more medicines of the same description given in combination frequently produce a much greater effect than the same dose of each individually would have done; and the influence exerted by one class of medicines upon those of a different character, not only generally but individually, is so infinitely varied, that it is almost impossible to say, *a priori*, what particular indications any such compound medicine is calculated to fulfil. It hence follows that we should be extremely cautious in excluding any medicine, however farraginous, the efficacy of which is supported by experience; and perhaps the old propensity for excessive complication in medicinal prescriptions, and for a vast choice of them, was not more reprehensible than is the modern one for excessive simplification and paucity. We know nothing of the specific properties of any medicine, whether simple or compound, but by its results; and we are no more justified in concluding that any composition is inadequate to effect the object for which it is administered, because its ingredients are respectively inert, than we should be in denying the violently explosive effect of gunpowder, because neither carbon, sulphur, or nitrate of potash, are severally possessed of that property. Nor is it unreasonable to suppose that a very slight variation of the ingredients of any compound medicine, or in their proportions, may sometimes make a considerable difference in its action. How can we tell, for instance, what new chemical compounds result from the mixture of ipecacuanha, opium, and sulphate of potass, in Dover's powder; or how the emetin, meconate of morphia, salt of potass, &c. mutually affect each other; and, if we cannot do so with respect to the compounds formed by artificially mixing different roots, gum resins, and salts together, still less can we do so with respect to those roots and gum resins themselves, composed as they are of numerous principles put together by the hand of nature; and this reflection should make us very jealous of rejecting certain of these principles, in the presumption that they are inert, and of relying upon others, in the presumption that they alone are active. The ridicule thrown upon multifarious medicinal prescriptions by Pliny among the ancients, and by Montaigne among the moderns, is perhaps deserved; and it is probably true that "one might as well," as Dr. Baynard remarks, "prescribe the powder of an old-fashioned bedpost as some of the receipts of ancient authors;" but it is to be feared that we have gone, or at least are going, too far at present on the simplifying system: "Præstat," says Cicero, "copiâ quam penuriâ premi." If Nicolaus Myrepsis has admitted into his work too many compound medicines, the number of which is not very far short of three thousand, and if Dr. Huxham was too fond of a complication of numerous ingredients in his prescriptions, which sometimes contained, it is said, not fewer than four hundred substances, it may be doubted whether we have not recently gone into the opposite extreme. The three British Pharmacopœias together do not contain in the present day more than fifty distinct compound medicines; but he who reflects as well on the endless diversities in the idiosyncrasies of the human body, as on the more or less specific action of every medicine, simple as well as compound, although he may not, like Van Mons, sigh for the restoration "to their primitive state of all those *bizarres* receipts whose credit time has spared," will yet certainly regret that the "*novæ vires mixturâ variorum*" of Gaubius, the new virtues of compounds, (which, as supposed by Dr. Ferriar, are analogous to the harmony of colours and of sounds,) the "*tertium quid*" of Shearman, or the adjective as well as substantive powers of Paris, should be sacrificed to a fastidious science, and that any compound medicine of acknowledged efficacy should ever be wantonly expunged and voted inert, because we cannot easily explain why it should be otherwise.

Before concluding these observations, I must observe, that they are deeply tinged,—particularly that portion relating to inflammation,—with the views taught and inculcated by my talented friend, Dr. Fletcher, of Edinburgh. It is with much pleasure that I look back to the time I attended that gentleman's class, and with still more that I acknowledge how great has been the colouring his instructions have given to my own opinions and practice. The reader will find some of the preceding doctrines ably supported, in a probationary essay by Dr. Fletcher, printed in 1829; upon which I have not hesitated occasionally to draw.

*Dumfries,* 1834.

